# Supplementary material for: mTOR Contributes to the Proteome Diversity through Transcriptome-Wide Alternative Splicing
Source: Int J Mol Sci. 2022 Oct 17;23(20):12416. doi: 10.3390/ijms232012416 (PMC9604279; doi:10.3390/ijms232012416)
Supplement: Supplementary file 1 [file ijms-23-12416-s001.zip › ijms-1917567-supplementary.pdf]

Supplemental Figures

A

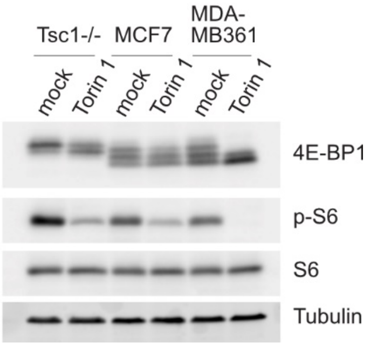

Figure S1. Cellular mTOR activity was different in *Tsc1*-/- MEFs, MCF7, and MDA-MB361. (**a**) Western blotting of downstream targets of mTOR (4E-BP1 and S6) in *Tsc1*-/- MEFs with DMSO or Torin 1 treatment (50nM, 24hr), MCF7 and MDA-MB361 cells with DMSO or Torin 1 treatment (100nM, 24hr).

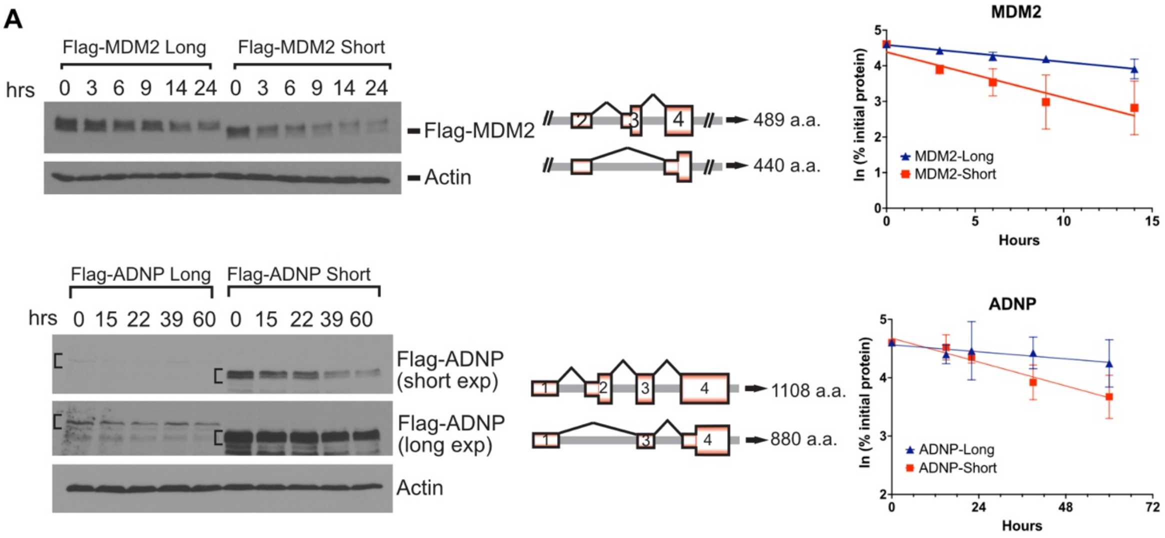

Figure S2. mTOR-driven exon skipping in MDM2 and ADNP showed differential protein stability trend. (**a**) Analysis of protein isoform stability by western blotting. Flag-tagged MDM2 and ADNP protein isoforms were transiently expressed in HEK293 and the difference in their stabilities was monitored in the presence of cycloheximide (30 ug/ml) for the indicated time points. Actin was used as a loading control. The protein level was quantified using densitometry in ImageStudioLite software and normalized to the actin level. Mean (SD) from two technical repeats were subjected to two-tailed Student's t-test for statistical analysis. P<0.05 as significant (*). n.s. denotes no significance.

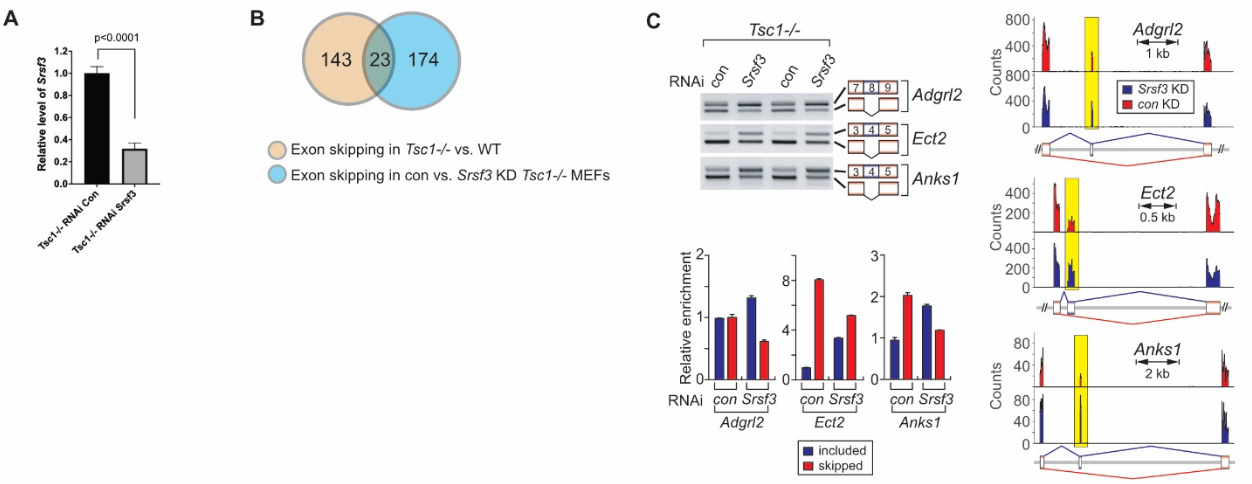

Figure S3. SRSF3 suppresses exon inclusion in mTOR-activated mouse embryonic fibroblasts. (**a**) qPCR quantitative expression analysis of *Srsf3* in the control and *Srsf3* siRNA knockdown *Tsc1*-/- MEFs. (**b**) Venn diagram illustrating the overlap of skipped exons in *Tsc1*-/- vs. WT MEFs and control vs. *Srsf3* knockdown in *Tsc1*-/- MEFs datasets. (**c**) RT-PCR and semi-quantitative gel electrophoresis of select transcripts with differential AS found in *Tsc1*-/- MEFs control vs. *Srsf3* knockdown treatment.
